# Promising cannabinoid-based therapies for Parkinson’s disease: motor symptoms to neuroprotection

**DOI:** 10.1186/s13024-015-0012-0

**Published:** 2015-04-08

**Authors:** Sandeep Vasant More, Dong-Kug Choi

**Affiliations:** Department of Biotechnology, College of Biomedical and Health Science, Konkuk University, Chungju, 380-701 South Korea

**Keywords:** Basal ganglia, Cannabinoids, CB1 receptors, CB2 receptors, Endocannabinoid signaling system, Neuroprotection, Parkinson’s disease

## Abstract

Parkinson’s disease (PD) is a slow insidious neurological disorder characterized by a loss of dopaminergic neurons in the midbrain. Although several recent preclinical advances have proposed to treat PD, there is hardly any clinically proved new therapeutic for its cure. Increasing evidence suggests a prominent modulatory function of the cannabinoid signaling system in the basal ganglia. Hence, use of cannabinoids as a new therapeutic target has been recommended as a promising therapy for PD. The elements of the endocannabinoid system are highly expressed in the neural circuit of basal ganglia wherein they bidirectionally interact with dopaminergic, glutamatergic, and GABAergic signaling systems. As the cannabinoid signaling system undergoes a biphasic pattern of change during progression of PD, it explains the motor inhibition typically observed in patients with PD. Cannabinoid agonists such as WIN-55,212-2 have been demonstrated experimentally as neuroprotective agents in PD, with respect to their ability to suppress excitotoxicity, glial activation, and oxidative injury that causes degeneration of dopaminergic neurons. Additional benefits provided by cannabinoid related compounds including CE-178253, oleoylethanolamide, nabilone and HU-210 have been reported to possess efficacy against bradykinesia and levodopa-induced dyskinesia in PD. Despite promising preclinical studies for PD, use of cannabinoids has not been studied extensively at the clinical level. In this review, we reassess the existing evidence suggesting involvement of the endocannabinoid system in the cause, symptomatology, and treatment of PD. We will try to identify future threads of research that will help in the understanding of the potential therapeutic benefits of the cannabinoid system for treating PD.

## Introduction

### Parkinson’s disease and cannabinoid system: prevalence and disease pathology

Parkinson’s disease (PD) is the second most common neurodegenerative disorder mainly affecting 1% of the elderly population [1]. In fact, age is the most critical risk factor for PD, and 1–2% of the world population > 65 yr suffers from this slowly progressive degenerative disease [2]. The preponderance of patients with PD have an idiopathic disease (90–95%) with no specific known cause, and the remaining have familial-inherited disease (5–10%) [3]. The exclusive hallmark feature of PD is the accumulation of α-synuclein protein, loss of dopaminergic neurons in the substantia nigra pars compacta (SNpc), which leads to depletion of dopamine in the striatum [4]. Several critical advances have been made in understanding the pathways that lead to cell dysfunction and death in PD. A few of these pathways include neuroinflammation, mitochondrial dysfunction, oxidative stress, kinase pathways, calcium dysregulation, protein aggregation, and prion-like processes. These pathways have helped to identify molecular targets [5], and based on experimental data, there has been a moderate increase in the tentative treatment choices for both early and later stages of PD and non-motor symptoms [6]. Although levodopa remains the primary mode of symptomatic treatment for PD, its chronic use is coupled with the development of motor complications such as response oscillations and levodopa-induced dyskinesia (LID), which affects 30–35% of patients after just 24 months of levodopa exposure. Thus, to avoid the motor complications arising with use of levodopa, on-going research pursues to develop new non-dopaminergic symptomatic agents capable to attenuate motor deficits and to restore dopamine transmission without producing dyskinesia [7]. Cannabinoids are one such interesting class of agents that not only have demonstrated neuroprotective ability but have also been evaluated for their potential to alleviate motor symptoms observed in PD. In this review we discuss the possibility of various cannabinoids and their respective target pathways that may hold potential to be used as an therapeutic for PD. Cannabinoids have been demonstrated to be effective in preclinical studies involving excitotoxicity, oxidative stress, neuroinflammation, and motor complications associated with PD [8].

### The endocannabinoid (endogenous cannabinoid) system

Considerable advances in our understanding of the elements of the ECB system have been made in the past 15 yr. Two major cannabinoid receptors, CB1 and CB2, have been cloned and several endogenous cannabinoids were identified along with their synthetic and degradative pathways. ECBs were discovered initially in the brain, but were also found in the periphery in humans and animals. ECBs are not only produced by cultured neurons [9], but also by microglia and astrocytes [10]. Years of research has identified few major ECBs such as arachidonoyl ethanolamide (anandamide, AEA), 2-arachidonoyl glycerol (2-AG), O-arachidonoyl ethanolamine (virodhamine), and 2-arachidonoyl glyceryl ether (noladin ether) [11]. As bulk of the investigations relating to ECBs and PD has considered data relating to AEA and 2-AG, we would majoritively consider these two ECBs in our further discussion. AEA is mainly been localized in the brain and periphery [12]. AEA, besides having activity on CB1 and CB2 receptors, also has full agonistic activity at TRPV1 receptor [13]. AEA is well distributed in the brain and shows high distribution in the hippocampus, thalamus, striatum, and brainstem and to a lesser extent in the cerebral cortex and cerebellum [14]. Lower concentrations of AEA are found in human serum, plasma, and cerebrospinal fluid [15]. Similarly, 2-AG is observed in both the brain and periphery, although its concentration is almost 150 times higher in brain compared to that of AEA [16,14]. Higher 2-AG levels are found in rat hippocampus, brainstem, striatum, and medulla [16]. 2-AG has greater potency, stability and agonistic activity at CB1 and CB2 receptors compared to that of AEA [17-19]. Two prominent areas involved in the control of movement, such as the globus pallidus and the substantia nigra, not only contain the highest densities of CB1 receptors [20] but also the highest levels of ECBs, specifically anandamide [21]. The physical composition of the nerve cells that yield ECBs in the basal ganglia is currently unknown, although the basal ganglia contain the precursor of anandamide and N-arachidonoyl phosphatidylethanolamine [22], which strengthens the theory of *in situ* synthesis for this ECB. Synthesis of anandamide seems to be related to dopamine. This hypothesis was backed by Giuffrida et al., who demonstrated that anandamide synthesis is regulated by dopaminergic D2 receptors in the striatum [23], suggesting that the ECB system acts as an inhibitory feedback mechanism countering the dopamine-induced facilitation of motor activity [23].

### Synthesis and metabolism of endocannabinoids

Distinct synthesizing and metabolizing enzymes have been identified, which actively regulate the levels of endogenous cannabinoids under normal and diseased conditions, and hence may be considered promising therapeutic targets. Both AEA and 2-AG are synthesized by cleavage of plasma membrane phospholipids, and calcium acts as a biosensor to depolarize the membrane to induce synthesis in an activity-dependent fashion [24]. AEA is synthesized by sequential actions of two intracellular enzymes, such as N-acyl phosphatidylethanolamine-specific phospholipase D (NAPE-PLD) that catalyzes the release of anandamide by a phospholipase D from its precursor N-arachidonoyl phosphatidylethanolamine and N-acyltransferase that catalyzes the transfer of arachidonic acid to a molecule of phosphatidylethanolamine to generate the precursor [24]. In addition to these enzymes other enzymes, such as protein tyrosine phosphatase, non-receptor type 22, and α/β hydrolase 4, are also involved in AEA production [25]. 2-AG is synthesized via three major pathways. The first pathway involves sn-1-diacylglycerol lipase α and β-mediated pathways [26,27]. Second pathway works via action of phospholipase-A1 to convert phosphatidyl lipid to 2-arachidonoyl lyso phosphatidyl lipid and then to 2-AG by the action of lyso-phospholipase-C. The third pathway includes hydrolysis of lipid phosphate by an lipid phosphate phosphatase [27]. Degradation of ECBs occurs rapidly *in vivo* [28-30]. FAAH is the predominant ECB metabolizing enzyme located intracellularly on post-synaptic neuron membranes [31-33]. FAAH is primarily responsible for breakdown of AEA, although 2-AG also acts as a substrate [33-35]. Monoacylglycerol lipase (MAGL) is a pre-synaptically localized enzyme that primarily inactivates 2-AG through hydrolysis to arachidonic acid and glycerol [36,37]. Apart from FAAH, acyl glycerol kinase [38], serine hydrolase α-β-hydrolase domain 6/12 (ABHD6/12), lipoxygenase [39] and cyclooxygenase 2 (COX-2) also have roles to metabolise ECBs [24]. Although, COX-2 can only be considered as an alternative metabolic pathway addressed to the synthesis of novel bioactive lipids rather than a central degrading pathway. All these new metabolising enzymes produce different molecules like, prostaglandin glycerol esters, lysophosphatidic acid and hydroperoxy derivatives of 2-AG. These by-products often have antagonizing role as compared to 2-AG. Therefore, impeding these metabolic enzymes may also act as a therapeutic target [27]. ECBs are lipophilic molecules and hence are capable of passing through the plasma membrane if their intracellular concentration is less than their extracellular concentration. However, crossing the plasma membrane as a mechanism for inactivation is too slow a process. Thus, the AEA membrane transporter (AMT) is a protein proposed to facilitate diffusion of 2-AG inside cells. Although AMT has not been isolated or cloned, its existence remains debated. However, reports have established cellular uptake of virodhamine [40] by AMT.

### Cannabinoids: role in neuroprotection and control of motor functions in PD

Cannabinoids have been contemplated as clinically neuroprotective molecules, as they can reduce oxidative injury, excitotoxicity, and calcium influx [41]. They also decrease inflammation by modulating glial processes that are associated with neuronal survival. Cannabinoids may provide neuroprotection in PD by means of these processes. Two important neuroprotective mechanisms are elicited by cannabinoids in experimental models of PD. First, they decrease increased oxidative stress in PD, a mechanism that seems to be independent of any involvement of cannabinoid receptors. Second, they increase density of CB2 cannabinoid receptors, mainly in reactive microglia, which regulate micro-functions of glial cells and homeostasis of surrounding neurons [42]. The basal ganglia is a part of a complex neuronal network that coordinates activity from different cortical regions that directly or indirectly participate in the control of movement [43]. Structural elements of basal ganglia include the corpus striatum and other subcortical regions such as subthalamic nucleus (STN), the substantia nigra and the pedunculopontine tegmental nucleus [43]. Historic and new data have empowered the notion of a marked role for the endocannabinoid (ECB) signaling system in the control of movement. This discovery is backed by three important lines of evidence. First, there is a marked presence of CB1 and CB2 receptors with vanilloid TRPV1 receptors coupled with ECBs in the basal ganglia and cerebellum, which are the areas that control movement. Second, there is evidence for a powerful inhibitory action of plant-derived, synthetic and endogenous cannabinoids on motor activity by fine tuning the activity of various classical neurotransmitters. Third, prominent changes take place in transmission of ECBs in the basal ganglia of humans and in animal models of PD. These lines of evidence strengthen the idea that cannabinoids act on key pathways of ECB transmission including receptors, transporters, fatty acid amide hydrolase (FAAH), which might be of therapeutic interest because of their potential to mitigate motor symptoms [44]. Considering the appropriateness of this preclinical evidence and the lack of efficient therapeutic strategies for PD, we will reassess the components of the ECB system with respect to their involvement in neuroprotection and alleviating the motor dysfunction associated with PD. We will also provide support for the hypothesis that modulators of the ECB system may have therapeutic potential for treating PD.

### Neuroanatomical basis for location and interaction between basal ganglial units and cannabinoid receptors

The molecular identification of the CB1 and CB2 receptors, the ion channel TRPV1, with their respective endogenous ligand systems has opened a whole arena of pharmacological effects elicited by each one of these specific receptor targets. CB1 and CB2 receptors belong to the superfamily of G protein-coupled receptors, which are coupled to inhibitory G proteins [30,45,46]. As such, both receptors inhibit adenylyl cyclase and activate mitogen activated protein kinase (MAPK) [47]. Moreover, presynaptically located CB1 receptors inhibit N and P/Q-type calcium channels and activate inwardly rectifying potassium channels [48-50]. Additional signaling mechanisms encompass focal phosphatidylinositol-3-kinase, adhesion kinase, sphingomyelinase, and nitric oxide synthase (NOS) [51-55]. The cDNA for CB1 receptor was first isolated from a rat cerebral cortex library using an oligonucleotide probe resulting from a member of G-protein-coupled receptors [45]. CB1 receptors are most highly expressed on axons and nerve terminals, but substantial functional evidence also confirms their expression on somata [47,56]. Autoradiography investigations have convincingly reported that the basal ganglia encompass the highest levels of both mRNA expression and binding sites for the CB1 receptor [57,58]. Including striatum [59], other three regions that receive striatal efferent outputs, such as the globus pallidus, entopeduncular nucleus, and substantia nigra pars reticulata (SNpr), contain high levels of CB1 receptor binding sites [20,60,61]. However, CB1 receptor mRNA transcripts are also present in the caudate-putamen, which is deficient of striatal outflow nuclei [62]. This observation agrees with the concept that CB1 receptors are presynaptically located in striatal projection neurons, a belief that has been backed by a series of anatomical experiments in which specific subpopulations of neurons in the basal ganglia were lesioned [63,64]. CB1 receptors are positioned in striatonigral (direct striatal efferent pathway) and striatopallidal (indirect striatal efferent pathway) projection neurons [59,65], which use gamma-aminobutyric acid (GABA) as a neurotransmitter. Glutamic acid decarboxylase, prodynorphin, substance P, as well as D1 or D2 dopaminergic receptors are other markers co-expressed in these pathways [59,66]. In contrast, intrinsic striatal neurons, which contain acetylcholine or somatostatin, do not express CB1 receptors [66]. Axon terminals and post-synaptic dendrites in the prefrontal cortex that express CB1 receptor are documented to have sub-cellular presence of D2 receptors [67]. Real-time PCR assays and quantitative autoradiography binding study demonstrated higher levels of cannabinoid receptor binding in the lateral globus pallidus and weaker CB1 receptor gene expression in the prefrontal cortex [68]. mRNA and autoradiographical studies revealed that the CB1 receptor is predominantly expressed in the sensory motor sectors of the striatum, with minor to minimal expression in associative/limbic striatal regions [69]. CB1 are localized both pre and post-synaptically. CB1 receptors are localized in GABAergic terminals of interneurons or collaterals from medium spiny neurons (MSNs), and also in glutamatergic but not in dopaminergic terminals Post-synaptically, CB1 receptors are localized in the somatodendritic area of MSN [70]. More extensive but less vigorous pre and post-synaptic CB1 receptor occurrence by electrophysiological and electron microscopic studies was also displayed in many brain regions including those enriched in dopaminergic neurons [71]. Thus displaying that the CB1 receptor is a significant retrograde signaling molecule in excitatory as well as in inhibitory-type axon terminals. Immunohistochemical, immunoblot [72] and autoradiographical studies have suggested the presence of CB1 receptor in substantia nigra, striatum and globus pallidus [73]. CB1 receptor immunolabeling is also abundant in SNpr [74]. Immunolabeling study by Matyas et. al; demonstrated that glutamatergic and GABAergic axon terminals in ventral tegmental area [75] and substantia nigra express CB1 receptor that target tyrosine hydroxylase containing dopaminergic projection neurons [76].

Another location for CB1 receptors in the basal ganglia is the subthalamopallidal and/or subthalamonigral glutamatergic terminals, as shown by the presence of measurable amounts of CB1 receptor mRNA in the subthalamic nucleus, coupled with the absence of detectable levels of cannabinoid receptor binding in that structure [62,77]. These anatomical studies and data strengthen the assumption that CB1 receptors play an important role in mediating the motor effects of various cannabinoid receptor agonists [78-80]. CB1 receptor expression is only partially known in glial cells. Microglia, astrocytes, and oligodendrocytes have been reported to express CB1 receptors [81,82]. CB1 immunoreactivity has been reported in perisynaptic and perivascular astrocytes [83] of rat striatum [84]. However, these data have not been reproduced by other researchers [85,86]. In addition, another study reported CB1 expression in primary astrocyte cultures and in astrocytoma cell lines [87,88]. Although CB1 expression on glial cells is debated, it is at a considerably lower density than that observed on neurons [47]. Primary microglial cell cultures express CB1 receptors. However, CB1 receptor expression is affected due to culture-influenced morphological changes that occur in microglia [81]. Thus, it is unclear whether the presence of CB1 receptors on microglia is a consequence of their activation, as there are no *in vivo* data regarding CB1 receptor expression in microglia. Carrier and colleagues reported that a non-transformed rat microglia cell line expresses CB1 receptors [89]. The functional relevance of CB1 receptors in microglial function remains dubious, although their role may be linked to nitric oxide (NO) production [54], which is a pivotal event in microglia-mediated neuroinflammation [90].

A second cannabinoid receptor was discovered in a human promyelocytic cDNA library within a few years following discovery of the CB1 receptor. Based on its homology to the CB1 receptor and similar ligand binding profile, this receptor was named the CB2 receptor [46]. There has been uncertainty with CB2 receptor expression on neurons. Some evidence described CB2 receptor expression in rat dorsal root ganglion (DRG) cultures [91,92] and F-11 cells that exhibits several features of authentic DRG neurons [93]. Related reports also indirectly displayed CB2 receptors on primary sensory neurons [94]. Furthermore, the localization of CB2 receptors in granules and purkinje cerebellar neurons of mouse brain was demonstrated [95,96]. CB2 receptors are primarily expressed on immune cells. Prolific expression of CB2 receptors is found in B-lymphocytes, natural killer cells, monocytes, neutrophils, T8 lymphocytes, and T4 lymphocytes [97]. Several other researchers have demonstrated the presence of CB2 receptors in *in vitro* cultures [98]. Specifically, mouse [99], primary rat [100-102], human [103], BV-2 [104], and RTMGL1 [89] microglial cells have been established to express CB2 receptors. Notably, CB2 receptor expression is affected due to culture-influenced morphological changes that occur in microglia [89]. However, data obtained by Benitoa and Nunez et al., indicate that CB2 receptors may be present in the perivascular microglial cells in normal pathologic human brains (cerebellum) [105,106]. Recent evidences through anatomical localization, behavioural studies for central effects of CB2 receptor agonists and mRNA expression profile in neurons are pointing towards the neuronal expression of CB2 receptors [107] in GABAergic neurons of layer II and V of medial entorhinal area of rats [108] as well as in CA1 hippocampus and substantia nigra [109]. Lately a report demonstrated the expression of CB2 receptors intracellularly in layer II/III pyramidal cells of the rodent medial prefrontal cortex [110]. Within the basal ganglia, CB2 receptors are also found to be expressed in neurons from both segments of the globus pallidus of *Macaca fascicularis* [111] and SNpr region of neonatal rats [112]. Immunohistochemical evidence suggests that activated microglia are able to express the CB2 cannabinoid receptor during chronic degenerative processes [113]. Past reports have also shown CB2 expression in neural progenitor cells [114]. These results indicate that CB2 might be involved in the neuroinflammatory process that develops in some forms of neurodegeneration such as PD [42]. Thus, various studies have established that CB2 receptors are significantly induced in different parts of brain, including the basal ganglia, in response to different types of insults including injury or inflammation [105,115]. Therefore, CB2 may become a promising target for modulating neuroinflammatory responses, as the agonists for this receptor are devoid of psychoactive effects [81,116]. The gist of these data is that immunocytochemical experiments claiming CB2 expression are several, but are often inconsistent. Also, before accepting any claim of CB2 expression in a specific tissue, the inclusion of coexisting and cautious controls is obligatory. Although these settings have been followed for many immune cells and neurons, but still many other tissues are yet to be established [116].

Some reports have also stated the importance of vanilloid TRPV1 receptors in the basal ganglia and their ability to interact with ECBs [117-119]. TRPV1 receptors have been studied for their role as molecular integrators of nociceptive stimuli present abundantly on sensory neurons. Apart from sensory neurons, TRPV1 receptors are also found in the basal ganglia circuitry co-localized with tyrosine hydroxylase, indicating that they are located in dopaminergic neurons of the nigrostriatal pathway [117,120]. Various neurochemical and pharmacological studies have reported the involvement of these receptors in the control of motor function [121] as well as in the manifestation of motor effects by certain cannabinoid receptor agonists [122]. Despite several reports in which these classical cannabinoid receptors do not explain the observed pharmacology, many studies using mice with genetically deleted CB receptors have confirmed the existence of additional targets, which are collectively known as non-CB1/CB2 receptors. Despite aggressive research efforts, the molecular identity of these non-CB1/CB2 receptors remains unclear [123]. The orphan G-protein-coupled receptor 55 (GPR55) has been discovered as another possible cannabinoid receptor [124]. In comparison to CB receptors, GPR55 is coupled to Gq, Gα12, and Gα13 proteins [125]. Regardless of high GPR55 expression in the striatum [126], contrasting pharmacological data may not consider GPR55 as a novel cannabinoid receptor [125,127,128].

### Pre-clinical and clinical manifestations displaying involvement of cannabinoids in PD

The outcomes of various investigations on the status of the ECB system in PD patients indicates a similar trend as that observed in animal models of PD. The cannabinoid signaling system in PD becomes over activated in the basal ganglia [129-133]. Impacts of cannabinoids on motor activity depend on the effect of the cannabinoid on the dopaminergic system. Systemic administration of endogenous [(AEA) & tetrahydrocannabinol (THC)] and synthetic cannabinoids [WIN-55,212-2, CP 55,940] typically inhibit motor activity and produce catalepsy in rodents [122,134-138]. A recent study has revealed a reduction in the availability of CB1 receptors in the SN of PD patients as compared with healthy controls [133]. Mice deficient of CB1 receptors display less severe dyskinesias, when lesioned with 6-hydroxy dopamine (6-OHDA) and treated with levodopa, compared with normal animals [139] thus, providing an indication for the participation of CB1-related mechanisms in motor regulation [140]. Electrophysiological studies have revealed that cannabinoid agonists increase the firing rate of SNpc neurons [141-143]. Mice lacking the CB1 receptor display a reduction in tyrosine hydroxylase-labeled varicosities, the majority of which are dopaminergic, in the accumbens shell as well as a loss of dopamine-dependent reward function [144]. *In vivo* microdialysis investigations have shown improved dopamine release in the striatum after administration of endogenous or exogenous cannabinoid agonists [145,146]. Patients with PD have increased efficacy of CB1 receptor activation along with an escalated binding of the CB1 receptor [129]. In addition, the cerebrospinal fluid of untreated patients with PD has augmented levels of AEA [147]. The outcome of a study involving the pattern and rate of cannabis use in almost 400 patients with PD revealed that 25% of patients had consumed cannabis and 45.9% proclaimed benefits [148]. Notably, one of the key symptoms of PD, bradykinesia, was most frequently ameliorated by cannabinoids, which is followed by muscle rigidity and tremor [148]. Moreover, use of cannabis mitigates dyskinesias in 14% of patients with PD [148]. In a short pilot study involving patients with PD, nabilone, a cannabinoid receptor agonist significantly decreased LID [149]. However, a larger, double-blind, randomized, placebo-controlled crossover trial demonstrated that orally administered cannabis extract was ineffective for alleviating parkinsonism or dyskinesia in patients with PD [150]. With respect to the neuroanatomical distribution, functional and behavioral studies, it suggests that the ECB system can act as an indirect modulator of dopaminergic neurotransmission in the basal ganglia which involves CB1 receptor mediated inhibition of GABA transmission. Another double-blind, randomized, placebo controlled study investigated the probable effects of antagonizing CB1 receptors in patients with PD, wherein progress in motor function or a decrease in LID was observed [151]. In another experimental randomized, double-blind, placebo-controlled trail, the CB1 receptor antagonist SR 141716 was ineffective for improving parkinsonian motor disability [151]. These discouraging outcomes indicate the necessity for more research in this area. Various facets concealing the relative “inadequacy” of these examinations might be different and probably are associated with the complex neuroanatomical network of the basal ganglia. Therefore, based on the above observations, we propose that a few CB1 receptor-related effects could be favored as compensatory mechanisms, whereas others effects might embody a part of the pathogenetic process, an issue that is further complicated with chronic L-DOPA use.

### Basal ganglial circuitry in PD and signaling of endocannabinoids

Heterogeneous loss of dopaminergic neurons in the SNpc and their projecting fibers in the striatum are the core pathological features of PD. The striatal nucleus is the main input area to the basal ganglia, as it gathers and holds glutamatergic cortical inputs from all operative sub-sections of the neocortex and a remarkable input straight from the thalamic nuclei. The striatal network, which consists of GABAergic projecting MSNs contributing to the sole striatal output, and cholinergic interneurons carry out the neuronal signal processing functions from the cortex [59]. Two sets of neuronal circuits exist for striatal MSNs that connect to the output nuclei of the basal ganglia. One is a direct circuit (direct pathway) or via a sequence of connections that include the STN and the external segment of the globus pallidus (GPe) (indirect pathway) [152]. The output nuclei [SNpr and the internal segment of the globus pallidus (GPi)] connect to the thalamus, which further has efferent extensions that form the cortico-basal ganglia-thalamo-cortical loop [152]. The physiological effect of dopamine originating from the SNpc on MSNs is intricate and not fully revealed. In fact, the intensity of membrane depolarization on the dopamine receptor dictates the type of effect produced. D1 dopamine receptors are positively coupled to adenylyl cyclase; hence, their activation increases the cytosolic cAMP level and subsequently elicits numerous downstream effects including an increase in NMDA receptor-mediated currents. In contrast, D2 dopamine receptors are negatively coupled to adenylyl cyclase and their activation decreases neuronal excitability and neuronal feedback to glutamatergic inputs [153].

**Figure 1:**
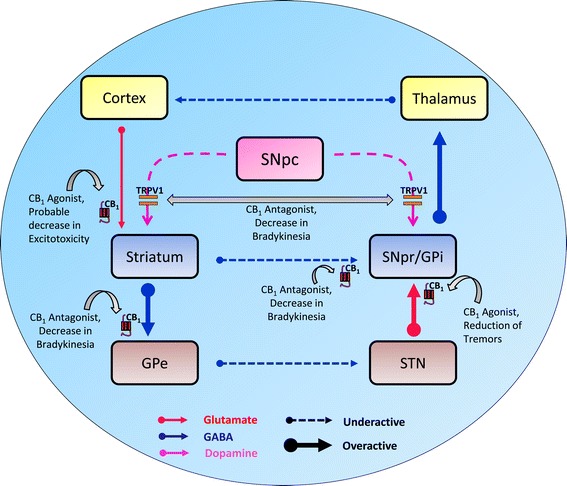
**Basal ganglial circuitry in Parkinson’s disease (PD) and tentative cannabinoid targets to improve motor disability in PD.** Progressive loss of dopaminergic innervation in PD causes overactivity of the indirect (inhibitory) pathway, resulting in excess glutamatergic drive to the GPi and SNpr and diminished activity of the inhibitory GABAergic direct pathway, further disinhibiting the activity of the GPi and SNpr. As output nuclei (GPi and SNpr) use the inhibitory neurotransmitter GABA, this amplified basal ganglia output leads to extreme inhibition of the motor thalamus which acts as a “brake” on motor activity; thus, resulting in the onset of parkinsonian syndrome. The neural circuitry above depicts various possible cannabinoid- based targets (CB1, CB2, and TRPV1 receptors) that can be used to mitigate the symptoms observed in PD. **Abbreviations:** CB1, cannabinoid receptor 1; TRPV1, transient receptor potential vanilloid 1; GPe, external segment of the globus pallidus; GPi, internal segment of the globus pallidus; SNpc, *substantia nigra pars compacta*; SNpr, *substantia nigra pars reticulata*; STN, subthalamic nucleus; GABA, gamma-aminobutyric acid.

Based on the classical hypothesis, MSNs in the direct pathway principally contain D1 dopamine receptors, whereas MSNs in the indirect pathway contain D2 dopamine receptors. Flow of dopamine through these two pathways produces opposite motor effects and thereby modulates activity of output nuclei that is thought to be essential for normal motor function. In fact, when a specific set of striatal neurons are triggered, repression of a subpopulation of pallidal neurons occurs which further clears the tonic inhibition from a specific target motor center, thereby initiating a motor reflex [59,154]. The continuous demise of pigmented dopaminergic neurons that occurs in PD decreases striatal levels of dopamine and creates an imbalance between the direct and the indirect basal ganglia pathways. This imbalance leads to over activity of GPi, which results in over-inhibition of the motor thalamus [155]. Over-inhibition of the motor thalamus reduces activity of motor cortex resulting in the onset of parkinsonian syndrome [155] (Figure 1). Recently, numerous levels of cross-talk between direct and indirect pathways have been discovered. As a result, a first level of interaction is represented by the molecular cross-talk between heteromeric D1and D2 receptors [156,157]. Activation of D1/D2 heteromers are demonstrated to mediate mechanisms like, increased intracellular Ca^2+^ levels, activation of calcium/calmodulin-dependent protein kinase II (CaMKII) and release of brain-derived neurotrophic factor (BDNF) [156,157]. These mechanisms are prerequisite for striatal physiology and LID [158,159]. A series of electrophysiological, biochemical, and anatomical experiments established that the constituents of the ECB system are markedly expressed at different levels in the basal ganglia neural circuitry and thus critically organize motor function and plasticity [160-162]. Dendrites and their presynaptic axon terminals of MSNs that innervate the GPi/e and the SNpr express CB1 receptors [160,161,163]. CB1 receptor expression is also found on corticostriatal excitatory glutamatergic terminals as well as in the excitatory extensions from the STN to the GPi/SNpr and SNpc [160,161,163]. Recent report is also supporting a role for the malfunctioning of corticostriatal glutamatergic signaling in the occurrence of LID [164]. Compared to the canonical neurotransmitters mentioned above, ECBs function as retrograde synaptic messengers. Retrograde signaling is the primary mode by which ECBs facilitate short and long-term forms of plasticity both at excitatory and inhibitory synapses and interacts with dopaminergic system [165,166]. The release and reverse journey of ECBs from postsynaptic neurons activates CB1 receptors located on presynaptic axons and thus decreases the release of neurotransmitter [167]. In fact, stimulation of presynaptic CB1 receptors on corticostriatal terminals decreases glutamate release [160,161]. Similarly, stimulation of CB1 receptors in the output segment of basal ganglia antagonizes both glutamate release from STN afferents and GABA release from striatal afferents [160,161,163].

Stimulation of presynaptic CB1 receptors in the GPe may increase local GABA levels by decreasing GABA reuptake from striatal afferents to this nucleus [160,161,163]. Dopaminergic signaling is bi-directionally linked to ECB signaling within the basal ganglia. In fact, D1 and D2 dopamine receptors are co-localised with striatal CB1 receptors on GABAergic neurons of striatonigral and striatopallidal pathways [58,160,161,163,166]. A tentative interaction between CB1 and D1/D2 receptors at the level of the G-protein/adenylyl cyclase signaling mechanism has been reported [23,60,168]. United activation of D1 and CB1 receptors causes a decrease in adenylyl cyclase and a net decrease in the inhibitory activity of direct striatal projection neurons ultimately leading to an inhibited motor response due to increased neuron activity in the SNpr. In contrast, co-stimulation of D2 and CB1 receptors increases adenylyl cyclase [59,60,169] which increases activity in the indirect striatal pathway that activates STN neurons leading to decreased motor activity [160,161]. This phenomenon of co-existence of macromolecular complexes composed of functional receptor units with biochemical properties that are different from those of its individual components is called receptor heteromers. Existence of CB1-D2 receptor heteromers was demonstrated using FRET study by Marcellino. et. al in 2008 [170]. In another study it was proposed that just by co-expressing CB1 and D2 receptors is adequate to induce stimulation of adenylyl cyclase in response to CB1 receptor activation [171]. The reasons for dissimilarities between these studies remains to be resolved, but all of these studies demonstrate that activation of CB1-D2 receptor heteromer can have completely opposite effects than activation of the individual receptors. Recent electron microscopy analysis with double labeling in the ventral striatum has established the presence of overlapping subcellular distributions of CB1 and D2 receptor immunoreactivities both at the pre and postsynaptic levels [172], providing significant support for the presence of CB1-D2 receptor heteromers in the striatum. Although some reports have suggested heterodimerization of CB1 and D2 receptors, [170,173] the functionality of these heteromers in striatal glutamatergic terminals has not been confirmed [70,174]. Apart from, CB1-D2 receptor heteromers, recently with the aid of biochemical and biophysical studies CB1-CB2 receptor heteromers is reported in nucleus accumbens and globus pallidus [175]. Typical characteristic feature observed with CB1-CB2 receptor heteromers is that, CB1 receptor antagonists blocks the effect of CB2 receptor agonists and, conversely, CB2 receptor antagonists blocks the effect of CB1 receptor agonists thus demonstrating a bidirectional phenomenon of cross-antagonism [175]. These heteromers may describe preceding conflicting results and may serve as therapeutic targets. Recent evidence suggests that dopamine modulates the activity of SNpc neurons not only by conventional dopamine receptors, but also by CB1 receptors, possibly via N-arachidonoyl-dopamine [176]. In addition to localization of CB1, the presence and functional role of TRPV1 on dopaminergic nigral neurons and their role in modulating synaptic transmission within the SNpc have also been determined [177]. TRPV1 immunostaining was observed in fibers and post-synaptically in striatal neurons [120], however the specific anatomical uniqueness of these TRPV1 expressing components has not been examined. It has been recently presented that CB1 and TRPV1 receptors decrease and increase the glutamate release from gliosomes [178] signifying a possible association of TRPV1 receptors in the regulation of cortical activity and plasticity. Additionally recent studies that establish the existence of different forms of TRPV1-mediated synaptic plasticity in the striatum [179], the presence of dissimilar forms of TRPV1-mediated cortical plasticity is highly probable, although this remains to be confirmed. Based on these reports, it is speculated that ECBs may critically regulate physiological functioning of the basal ganglia neuronal circuit. Additionally, the existence of elements of the ECB system in different neural circuits and their direct interaction with GABAergic, glutamatergic, and dopaminergic signaling systems makes these components an ideal non-dopaminergic target for PD.

### Consequences of corticostriatal plasticity in PD: role of endocannabinoids

Synapses, particularly those in the striatum, sustain long-lasting morphological and functional modifications after continuously activating neuronal pathways [158]. Synaptic plasticity seems to play an important role in the dynamics and development of a neuronal circuit in the corticostriatal region, particularly motor learning. Continuous stimulation of striatal synapses of MSNs in the corticostriatal pathway elicits both long-term depression (LTD) and long-term potentiation (LTP) of synaptic transmission efficacy. Activation of dopamine receptors is a prerequisite for both LTD and LTP at corticostriatal synapses [158,166]. This phenomenon is observed to be impaired in both the striatum and the motor cortex of patients with PD as well as in experimental models of PD [180,181]. In contrast, ECBs are actively involved in the formation of LTD synapses that connect striatal and cortical neurons and therefore plays a vital role modulating the dynamics of the striatal neural circuit. Elevated intracellular calcium, stimulation of D2 receptors and activation of striatal MSNs are reported to discharge ECBs such as AEA [23,28,59]. Hence, it is speculated that release of AEA from postsynaptic neurons under such circumstances might act as retrograde messenger stimulating presynaptic CB1 receptors and initiating long-lasting depression of excitatory glutamatergic transmission [182-184]. However, it has been hypothesized that MSNs of the direct and indirect striatal pathways might manifest diverse synaptic properties [174]. In particular, satisfactory release of ECBs requires ECB-mediated LTD which is limited only to MSNs in the indirect-pathway [174]. The role of ECBs in the control of LTD of MSNs is an important topic of discussion having great consequence on the direct/indirect pathway [59]. ECB-dependent synaptic plasticity of MSNs could depict a synaptic mechanism for the formation of persistent drug-related behaviors [59]. In agreement with this view, it has been witnessed in a mouse model of cannabinoid tolerance that continuous activation of the ECB pathway impairs LTD in MSNs [185]. Synapses between MSNs in the indirect-pathway are abolished in experimental models of PD [186,187]. This deficiency can be recovered by a D2 dopamine receptor agonist such as quinpirole or by URB597, an inhibitor of FAAH [174]. Administering URB597 and quinpirole significantly decreases catalepsy and increases locomotor activity in experimental models of PD [174]. This result indicates a direct interrelationship between recovery of ECB-mediated synaptic plasticity at corticostriatal synapses and improvement in PD motor symptoms. Also, within the striatum, sub-class of GABAergic interneurons that are observed to produce NO [59] and cholinergic interneurons are found to express CB1 receptors [188]. In line with these reports various electrophysiological experiments have also demonstrated that inhibitors of NOS avert induction of LTD [189,190]. Loss of LTD expression at glutamatergic striatal synapses on both the classes of MSNs has been reported to cause LID [189]. Therefore, damage to ECB-dependent striatal LTD at corticostriatal synapses may contribute to the abnormal activation of this specific neuronal circuit culminating in over stimulation of GPi and subsequent over-inhibition of the motor cortex leading to the initiation of parkinsonian syndrome.

### Therapeutic availability of cannabinoids for motor symptoms in PD

Cannabinoids were previously reported to only produce behavioral patterns such as catalepsy and hypolocomotion in experimental animals. Due to these peculiar behavioral effects of cannabinoids, their therapeutic use for alleviating bradykinesia, rigidity, and other hypokinetic symptoms typical of PD is limited [148,191,192]. These effects lead to an array of studies that investigated various facets of cannabinoids on motor symptoms in PD. The evidence obtained in different animal models and in clinical trials produced a basis for the involvement of cannabinoids in motor behaviors. As cannabinoids lack specificity of binding to the desired target, the data obtained varied in specific motor effects of cannabinoids but it also opened new doors for their clinical utility. The evidence that ECBs such as AEA not only bind to CB1 receptors but also bind to TRPV1 receptor, has diverted the focus of research into novel mediators that regulate motor effects of cannabinoid [193]. Therefore, the presence of ECBs in different regions of the basal ganglia circuitry along with the polymorphous nature of cannabinoid-mediated mechanisms makes it a complex physiological phenomenon eliciting behavioral effects. Many experiments aimed at outlining the effects of CB1 agonists and antagonists and their potential utility in PD (Figure 1), have produced inconsistent data, as there are many complex responses produced by dopamine and its interaction with different cannabinoid mechanisms [193].

Since CB1 receptors are highly expressed in both D1 and D2 receptor containing MSN, and they antagonize D1 and D2 receptor mediated behaviors, mounting evidences have suggested the involvement of endocanabinoid system in dyskinesia [58]. This is the central motive to hypothesize CB1 receptor as a therapeutic target to regulate the imbalance of glutamatergic or GABAergic neurons in PD and dyskinesia [194]. There is also a report that ECBs and cannabinoid agonists decrease dopamine reuptake by inhibiting dopamine transporters [195] and hence may have applications for fine tuning the striatal neuronal network involved in dyskinesia [196]. Initial studies concerning motor behavior involved investigating the effect of the cannabinoid agonists CP 55,940 and WIN-55,212-2 on rotational behavior induced by the D1 dopamine receptor agonist SKF 38393 in 6-OHDA model of PD [136]. Rotational behavior induced by SKF 38393 was mitigated by both cannabinoid agonists, but the same response was not observed when they were tested with the D2 agonist quinpirole. In contrast, WIN-55,212-2 decreased the effects of the D2 agonist, but not those of a D1 agonist in reserpinized rats [197]. Although there was a discrepancy in the effects elicited by the cannabinoid agonist on different dopamine agonists, these data demonstrate that cannabinoid agonists antagonize the effect of dopaminergic drugs. Consistent with the pharmacological model, the cannabinoid receptor antagonist rimonabant (SR 141716A) was found to boost the locomotive effects of quinpirole in normal and reserpinized rats and also increased the locomotor activity in mice pre-exposed to 9-THC [21,23,198]. However, paradoxical results were obtained by the SR 141716A in primate models [168]. Rimonabant was found to be unsuccessful [131] to antagonize motor deficits in 1-methyl-4-phenyl-1,2,3,6-tetrahydropyridine (MPTP)-intoxicated primates [130], although these experiments used different primate species. Also, in a confined clinical trial, rimonabant had no antiparkinsonian effects in combination with levodopa [151].

Blocking CB1 receptors may be effective only in particular circumstances, such as when low doses of CB1 receptor antagonists are used, when patients do not respond to dopamine therapy, or when they are in progressive phases of the disease [199-201]. Although the data obtained were from drugs with different specificities and in different animal models, these results indicate an indecisive effect of CB1 antagonists on parkinsonian symptoms. This result indicates a central advantage, as it may provide a novel anti-parkinsonian agent useful for circumstances in which classic dopaminergic replacement therapy is futile. The synergism of antiparkinsonian effects caused by cannabinoid antagonists with dopaminergic drugs to stimulate movement suggests that cannabinoid agonists may antagonize the actions of dopaminergic drugs, including LID. Long-term levodopa therapy for PD generally results in variations in motor responses called dyskinesias or abnormal involuntary movements (AIM) [202,203]. Few evidences supporting this hypothesis are cannabinoid agonist, WIN-55,212-2 that produced antidyskinetic effects in rodents [204], and nabilone that reduced dyskinesia in primate models and patients [149,205]. The effect of CB agonist depends on the fact that CB1 receptors are expressed on the presynaptic terminals of the striatonigral and striatopallidal neurons (GABAergic neurons), as well as on the presynaptic terminals of corticostriatal neurons (Glutamatergic neurons), and are thought to exert a tonic inhibitory effect via retrograde signalling from postsynaptic neurons (Figure 1). Therefore, it can be proposed that cannabinoid agonists may decrease dyskinesia by antagonizing the effects of dopaminergic drugs. Nonetheless, some evidence does not match the above hypothesis, as the selective cannabinoid antagonist rimonabant decreases LID in MPTP-treated marmosets [130]. In contrast, another study reported the ineffectiveness of the CB1 antagonist CE-178253 in parkinsonian rhesus monkeys [206]; thus, indicating a tentative role of animal species and behavioral outcome. However, clinical trials failed to reproduce the same effect using a cannabis extract; thus, questioning the true use and activity profile of cannabinoids [150,163]. In contrast, another study demonstrated amelioration of parkinsonian symptoms and dyskinesia after discontinuing use of cannabis for months with no clear explanation [148]. In conclusion, it can be stated CB1 antagonists seem to have antiparkinsonian effects (antidyskinetic effect), whereas activities of CB1 agonists appear to be highly ambiguous [193].

A very recent study by Mathur et. al; have reported the involvement of corticostriatal ECB-LTD in the development of LID [166]. Another report related to this study, demonstrated that, treatment with L-DOPA causes expression of ECB-LTD only in non-dyskinetic parkinsonian rats and not in dyskinetic rats [189]. Furthermore decreased levels of cGMP signaling in the brain were also observed in hemiparkinsonian rats with LID [207]. Specific inhibition of phosphodiesterase-5 by zaprinast and UK-343664 was found to restore ECB-LTD in these dyskinetic rats and mitigate the incidence of dyskinetic behaviors [189]. Thus, restoring ECB-LTD can be considered as a potential target for therapeutic intervention in PD. Apart from dopaminergic neurons, striatal serotonergic neurons are capable of using L-DOPA to release dopamine which contributes towards LID [208]. Agonist for serotonergic neuron autoreceptors (5-HT1a/b) are been speculated to abate corticostriatal glutamatergic release and thus show efficacy in mitigating LID [209]. This data agrees with the study done my Picconi et. al, wherein only L-DOPA treated rodents having LID elicit loss of ECB-LTD. Lastly, new data has suggested a role for the regulator of G-protein Signaling 4 (RGS4) protein in modulating ECB production in MSNs of indirect pathway. RGS4 is as a strategic link between D2/A2A signaling and ECB mobilization pathways. It was found that inhibition of RGS4 was observed to reinstate ECB-LTD in the presence of a D2-antagonist (sulpiride), while RGS4 deficient 6-OHDA denervated mice were resistant to some features of motor dysfunction typical of parkinsonism [210]. Based on these results, RGS4 can serve as a nondopaminergic target to treat PD. Cannabinoid-mediated mechanisms in the striatum play a crucial role regulating dopamine-induced motor behaviors. Activating CB1 receptors increases neuronal activity in SNpc [146]. This finding is opposed by a report wherein ECBs such as AEA and other related congeners acting through postsynaptic TRPV1 receptors may diminish nigrostriatal dopaminergic cell activity [122]. Nevertheless, other authors have stated a surge in dopamine release after stimulating TRPV1 receptors in the SNpc [177,211]; however, this improvement may be facilitated by TRPV1 receptors located in glutamatergic terminals in the SNpc rather than by receptors located in dopaminergic terminals.

Presynaptic CB1 receptors in corticostriatal terminals modulate discharge and uptake of glutamate; thus, causing a decrease in glutamate-mediated excitation in MSNs (Figure 1) [212]. Based on interacting dopaminergic mechanisms and their corresponding regulatory status, modulating glutamate transmission might result in different motor effects. Furthermore, coupling postsynaptic CB1 receptors with G-proteins has contrasting regulatory effects on D1 and D2-mediated responses, such as negative and positive regulation, respectively [168]. It is unclear whether the striatal reduction is due to a lesion or by an increase in ECBs as a compensatory mechanism [213] with respect to changes occurring after dopaminergic loss in the cannabinoid system [204]. Correspondingly, CB1 receptor binding also changes with the demise of dopaminergic neurons [129,213]. Some modulation by cannabinoids may occur due to the changes produced by dopamine deprivation during the early and preclinical stages of the disease, and this modulation becomes incompetent and motor symptoms develop as the disease advances [160]. Regardless of the controlling position of the striatal cannabinoid system in PD, pre and postsynaptic machinery mutually result in precise effects on isolated projection neurons contributing to the drug induced-behavioral changes. Thus, numerous factors intercede in the striatal activities of cannabinoid agonists and antagonists to regulate their effects on motor responses to dopaminergic drugs. The effects of CB1 agonists to attenuate LID may be facilitated by striatal machinery where the cannabinoid system is controlling a weakened dopamine system that pushes errors of activity (mistake-proofing) and discharges involuntary movements. Glutamate/NMDA antagonists may help to reinstate normal responses to levodopa with attenuation of dyskinesias [214-216] by decreasing the activity of glutamatergic projections, an action similar to CB1 agonists in the striatum.

Both the cannabinoid actions have been established by electrophysiological studies on the discharge of GABA and glutamate, although their communication may lead to specific synaptic transmission effects, and these effects remain unspecified. In addition, it is unknown whether one of these mechanisms dominates after dopaminergic loss to elicit a clear behavioral response, as they are functionally contrasting. Hallmark features of cannabinoids to increase GABA and reduce glutamate transmission strongly impede neuronal activity in GPe and result in catalepsy [217]. In line with this report, neurons containing D1 receptors participate in the motor depressant effects under the influence of presynaptic CB1 receptors while interaction of neurons containing D2 receptors with postsynaptic CB1 receptors are thought to mainly facilitate the cataleptogenic effects of cannabinoids [218]. Depleting dopamine levels significantly increases the levels of ECBs in GPe [21]. So, CB1 antagonists, via mechanisms in the indirect pathway, may synergize with the antiparkinsonian effects of levodopa. Moreover, CB1 receptors inhibit GABA release on presynaptic neurons in the STN [219], which may add to this synergism. Consequently, the cannabinoid activities in these areas of the indirect pathway are well-matched with the antiparkinsonian properties of CB1 antagonists and the antidyskinetic effects of CB1 agonists. The main caution is that these activities cannot be secluded from other vital cannabinoid actions in additional basal ganglia circuits. Existing data suggest no adequate proof to establish the mechanisms overriding the behavioral responses of cannabinoids administered systemically in a parkinsonian background. In addition, cannabinoid drugs with comparable abilities have elicited diverse motor responses even when they display high CB1 receptor specificity. Despite experimental disparities, incongruous evidence has been obtained from pre-clinical and clinical models, with drug doses acting on numerous sites of basal ganglia, suggesting a highly intricate mechanism wherein cannabinoids might bind to other locations and act through other mechanisms besides the extensive CB1 regulation of corticostriatal synapses controlling motor effects [193]. Crucial data have appeared from experiments suggesting that ECBs bind to other than CB1 receptors [220,221]. Thus, the practical role of additional binding sites becomes essential to the mechanisms responsible for cannabinoid effects [222].

Findings by Morgese and colleagues described the effects of cannabinoids via binding to dissimilar receptors in a LID parkinsonian model of rat [223]. The study involved dopamine-denervated rats, wherein increased levels of AEA were attained by administering the URB597. URB597 had noteworthy effects on levodopa-induced AIMs only if URB597 was co-administered with capsazepine, a TRPV1 antagonist. These results demonstrate that ECB binding to the TRPV1, antagonizes their antidyskinetic effects mediated presumably by binding to CB1 receptors [223,224]. The favorable effects of CB1 receptor antagonists against bradykinesia in PD may be due to the hyperkinetic ability displayed by blocking TRPV1 receptors (Figure 1). This hypothesis is based on the suggestion that stimulating TRPV1 receptors present on nigrostriatal dopaminergic neurons [117] impedes the synthesis and release of dopamine in striatal dopaminergic terminals [122]; therefore, their pharmacological blockade may produce the opposite effect and improve the motor inhibition characteristic in PD. Nevertheless, this approach might have a disadvantage in terms of a potential clinical application for TRPV1 receptors to lessen bradykinesia in PD, as advanced phases of PD are accompanied by death of nigrostriatal dopaminergic neurons and loss of TRPV1 receptors [225]. Multiple reports documenting different results nurture questions regarding the receptor binding profile of cannabinoid drugs that elicit contradictory results through frequent testing in animal models and clinical trials. AEA is one of the most studied ECBs. AEA is a partial agonist at the CB1 receptor and also binds to TRPV1 [220,121,226] as a partial agonist, by which few of its numerous pharmacological effects may be facilitated [227]. According to an electrophysiology study, vanilloid receptors control glutamate signaling to dopaminergic neurons in the substantia nigra [211]. Moreover, there is mutual expression of CB1 and TRPV1 receptors in areas of the basal ganglia, and preceding studies [228] have shown their functional communication with dopaminergic neurons. Interestingly, oleoylethanolamide (OEA) also augments the effect of AEA by acting on TRPV1; furthermore it decreases LID at doses that do not change motor behavior in the mouse model of dyskinesia [229]. This result is in agreement with another report wherein, capsaicin, a specific TRPV1 agonist, eliminated both the increase in molecular markers of dyskinesia and the anti-dyskinetic effects of OEA [196]. The communication between CB1 and TRPV1 receptors appears to be principally controlled by some key molecules such as FAAH [230]. The metabolic activities of FAAH and AMT in the striatum are abated in 6-OHDA lesioned rats [213]. Therefore, it is conceivable that motor variations can be initiated by potentiating AEA activity using an FAAH inhibitor. In comparison to AEA, WIN-55,212-2, a CB1 agonist with inhibitory effects on the TRPV1 receptor [231], has analogous effects on AIMs as those induced by increasing AEA by co-administration of a TRPV1 antagonist, as AEA is a partial agonist of both receptors. Also, treatment with a CB1 antagonist did not reverse the antidyskinetic effects of elevated AEA. Hence, the antidyskinetic effects of ECBs may be mediated by binding to receptors other than CB1. Therefore more than a few inferences can be drawn from these data. First, the binding locations facilitating the AEA induced motor responses remains to be established; thus, ECB receptors involved in motor activities consist of vanilloid and others anonymous receptors [232]. Second, the motor responses for the CB1 receptor agonist WIN-55,212-2 are also influenced by its activity at TRPV1 and other cannabinoid or additional cannabinoid sites. Lastly, mechanisms facilitated by cannabinoid binding to TRPV1 and possibly other vanilloid receptors are associated with motor responses to levodopa [193] and also suggest TRPV1 as a potential therapeutic target for PD which is devoid of dyskinesia [194].

### Neuroprotective prospective of cannabinoids in PD

Substantial bulk of information has been accumulated that suggests a strong potential for cannabinoid compounds that could provide neuroprotection against acute or chronic neurodegenerative disorders (Table 1) [132,138,233-236]. This information is mostly relevant considering the post-mitotic features of neuronal cells, in which repair processes are particularly difficult after several types of brain injuries. However, cannabinoids have also participated in the regulation of neurogenic reactions in definite structures of the brain [186,237]. The neuroprotective ability of cannabinoids has been displayed against acute neuronal degeneration as shown by diverse *in vitro* and *in vivo* models that reproduce cytotoxic events causing cell death [235,238]. Additionally, cannabinoids are also documented to elicit neuroprotection in progressive neurodegenerative pathologies involving mitochondrial dysfunction, inflammation, oxidative stress, and excitotoxicity [239-241]. Both CB1 and CB2 receptor are reported to be induced in response to brain damage and/or inflammation [132,239]. For instance, CB1 receptors were up-regulated after experimental stroke [242,243], and in response to excitotoxic stimuli in newborn rats [244]. As compared to CB1 receptor, most of the pertinent data was obtained with CB2 receptors owing to their negligible presence in brain [239,245,246]. CB2 receptors have been demonstrated to be modulated in various neurodegenerative diseases preferentially involving astrocytes and microglia in disease pathology of stroke [247], Huntington’s disease (HD) [115,248], in patients with Down’s syndrome, Multiple sclerosis (MS) and Amyotrophic lateral sclerosis (ALS) [249-251], in the senile plaques of patients with Alzheimer’s disease (AD) [105] and in pre-clinical model of AD and MS in rats [252,253]. Agonists of CB2 receptor protect against neuronal damage in pre-clinical models of focal ischemia [254], AD [255], HD [247,248], MS [253], and ALS [256]. From all these studies it was anticipated that neuroprotection conferred by agonists at CB2 receptors were associated to the presence of these receptors in glial components. However recent report has established that the activation of CB2 receptors located in neurons may also be defensive against remote-axotomy-induced apoptosis, an outcome that involves the activation of PI3K/Akt signaling [257]. This displays that various cannabinoid elements that are altered in response to neurotoxic insults in different experimental models of neurodegeneration can be targeted to develop a tentative therapeutic. However, the degree and accuracy of these alterations depend on several parameters, such as animal species, age, type and severity of injury, and mechanisms activated in the cell [258].

**Table 1:** **Summary of the pharmacological effects demonstrated by cannabinoids in various model of PD and other neurodegenerative diseases**

**Compound**	**Model**	**Activity profile**	**Ref.**
Oleoylethanolamide (OEA)	6-OHDA model of PD in mice	OEA reduces dyskinetic symptoms and molecular markers of dyskinesias including striatal overexpression of FosB and phosphoacetylation of histone 3	[196]
Oral Cannabinoid Extract (OCE)	A Class I double-blind crossover study in dyskinetic patients	OCE was ineffective for treating levodopa-induced dyskinesias in patients with PD	[334]
Cannabis administration via smoking	Open-label observational study in 22 PD patients	Cannabis was found to improve tremor, rigidity and bradykinesia in PD patients. Also, sleep and pain scores were also improved	[335]
WIN-55,212-2	L-DOPA-induced motor fluctuation model of PD	WIN-55,212-2 significantly reduced AIMs to L-DOPA in 6-OHDA-lesioned rats by modulating DARPP-32 and ERK1/2 phosphorylation in striatal neurons	[336]
OEA and Palmitoylethanol-amide (PEA)	LPS-induced neuroinflammation in rat	OEA and PEA inhibited oxidative and nitrosative stress by reducing LPS-induced NFκB expression and subsequent release of proinflammatory mediators	[337]
WIN-55,212-2 and HU-210	Intranigral injection of LPS in rats	WIN-55,212-2 and HU210 increased the survival of nigral neurons, inhibited activation of NADPH oxidase, ROS production and production of proinflammatory cytokines	[338]
THC	MPP^+^, lactacystin and paraquat induced neurotoxicity in SH-SY5Y cells	THC exhibited neuroprotective effect against all toxins probably by activation of PPAR-γ receptors	[339]
THCA, THC and CBD	MPP^+^ induced cytotoxicity to mice mesencephalic cultures	All cannabinoids exhibited anti-oxidative action. THC and THCA protected dopaminergic neurons	[340]
WIN-55,212-2	L-DOPA-induced (AIMs) in the 6-OHDA injected rat	WIN-55,212-2 ameliorated L- DOPA induced AIMs	[341]
WIN-55,212-2	PSI-induced cytotoxicity in PC12 cells	WIN-55,212-2 protects PC12 cells from PSI-induced cytotoxicity, Inhibits cytoplasmic accumulation of parkin and α-synuclein	[342]
WIN-55,212-2 and HU-210	MPTP model of PD	WIN-55,212-2 and HU210 increased survival of DA neurons in the SN, reduced expression of proinflammatory cytokines and improved motor function	[343]
(9)-THCV	Unilateral 6-OHDA lesions in rats	(9)-THCV attenuated the motor inhibition	[273]
(9)-THCV	LPS model of PD in mice	(9)-THCV decreased microglial activation and protected nigral TH neurons	[273]
AM251 and HU210	Levodopa-induced dyskinesia in a rat model	HU210 significantly reduced certain subtypes of AIMs while, AM251 had no effect on AIMs	[344]
WIN-55,212-2	MPTP model of PD	WIN-55,212-2 protected TH neurons in the SN	[42]
Rimonabant	Unilateral 6-OHDA lesions	Rimonabant improved motor behavior	[345]
JWH015	MPTP model of PD in mice	JWH015 reduced MPTP-induced microglial activation	[42]
Adenoviral vector enforced expression of the CB1 receptor	R6/2 mouse model of HD	Vector-enforced expression of CB1 receptor causes re-expression of BDNF and cures neuropathological deficits	[346]
CBD	3NP model of HD in rats	CBD protected striatal neuron by completely reversing 3NP-induced reductions in GABA contents and mRNA levels for SP, NSE and SOD-2	[347]
CBD	β-amyloid-induced model of AD in rats with or without GW9662	Presence of GW9662 was able to significantly block protective effects of CBD on reactive gliosis and on neuronal damage. CBD also induced hippocampal neurogenesis	[280]
JWH-133	AβPP/PS1 genetic model of AD	JWH-133 lowered microglial activity, decreased expression of pro-inflammatory cytokines and tau hyperphosphorylation	[348]
Sativex®	Human tau overexpressing mice model of AD	Sativex® decreased gliosis and generation of free radical in hippocampus and cortex	[349]
MDA7	Aβ-induced model of AD in rats	MDA7 mitigated the expression of microglia and astroglial markers, reduced the secretion of interleukin-1β, diminished the increase of CB2 receptors, promoted clearance of Aβ and restored synaptic plasticity, cognition, and memory	[350]
CBG	3NP model of HD in mice	CBG improved motor deficits and preserved striatal neurons. CBD also decreased reactive gliosis and upregulated antioxidant defenses	[351]
HU210	PC12 cells model of HD expressing mutant huntingtin	HU210 increased cell survival, by cyclic adenosine monophosphate and extracellular signal-regulated kinase mechanisms	[352]
ACEA, HU-308 and CBD	Malonate induced model of HD in rats	Activation of CB2 receptor diminished reactive gliosis and subsequent release of proinflammatory cytokine	[115]

***Abbreviations:*** DA, dopamine; THCA, Tetrahydrocannabinolic acid; CBD, cannabidiol; MPP+, 1-methyl-4-phenylpyridinium; AIMs, abnormal involuntary movements; SN, substantia nigra; 9-THCV, tetrahydrocannabivarin, THC, Tetrahydrocannabinol TH, tyrosine hydroxylase; LPS, lipopolysaccharide ; 6-OHDA, 6-hydroxydopamine; PSI, proteasome inhibitor; 3NP, 3-nitropropionic acid; HD, Huntington’s Disease; SP, substance P; NSE, neuronal-specific enolase; SOD, superoxide dismutase; AD, Alzheimer’s disease; MDA7, 1-((3-benzyl-3-methyl-2,3-dihydro-1-benzofuran-6-yl) carbonyl) piperidine; CBG, Cannabigerol.

### Therapeutic role of cannabinoids in oxidative stress in PD

Augmented oxidative stress has long been linked with PD [259]. Reactive oxygen species (ROS) derived from mitochondria are involved in PD pathology as weakened mitochondrial function and increased oxidative markers cause neuronal injury in PD patients [260]. Cellular and animal studies have emphasized a role for superoxide anion produced by microglial NADPH oxidase in augmenting the demise of dopaminergic neurons in PD [261]. Recently low levels CB1 receptors have been detected on mitochondrial membrane hinting towards a direct relationship between CB1 receptor and mitochondrial functions in the brain [262]. The phenolic ring moieties in cannabinoids [263] have been found to display antioxidant activity and guard against glutamate-induced neurotoxicity in a cellular model [264]. More recent evidence in rodents indicates that cannabinoid treatment might protect against neuronal damage in diabetic neuropathy [265] and the cognitive impairment induced by experimental sepsis [266]. One of the latest studies documented the protective effects of synthetic cannabinoids on paraquat-induced generation of mitochondrial ROS [267]. Taken together, these discoveries support the hypothesis that treatment with cannabinoids having antioxidant effects may modulate mitochondrial ROS production [56] in the PD brain. Apparently, cannabinoids can also quench ROS generated by microglial NADPH oxidase in the extracellular space that cause neuronal damage, but this hypothesis has not been tested yet. A study in C57BL/6J mice with cisplatin-induced nephropathy established that the antioxidant cannabidiol diminishes the increase in NADPH oxidase expression and decreases markers of inflammation, oxidative stress, and cell death in kidneys [268]. The mechanism by which cannabidiol acts to reduce NADPH oxidase expression and inhibit oxidative injury within the PD brain has yet to be confirmed but it seems to act through mechanisms independent of CB1 or CB2 receptors [44].

This finding is specifically important, as hypokinetic activity of cannabinoids that stimulate CB1 receptors signifies a drawback for PD because such compounds acutely augment rather than diminish motor disability [138]. Consequently, major efforts are being focused at exploring cannabinoid molecules that deliver neuroprotection through their antioxidant properties and which specifically activate CB2 receptors and may even antagonize CB1 receptors to provide additional benefits for alleviating symptoms such as bradykinesia [44]. Nonetheless, other evidence also supports the hypothesis that cannabinoids may confer protection against PD pathology in rodent models due to their antioxidant properties. Synthetic cannabinoids with antioxidant abilities protect against pathological features encountered in rats injected with 6-OHDA [225,269]. AM404 and cannabidiol, which have validated anti-oxidant activity in cellular models but slight to no affinity for CB receptors [270], have established neuroprotective effects. Although the direct effects of cannabinoids on indicators of oxidative stress were not measured, treatment with cannabinoid did avert a decrease in cytosolic mRNA levels of endogenous anti-oxidant copper-zinc superoxide dismutase following 6-OHDA intoxication [269].

**Figure 2:**
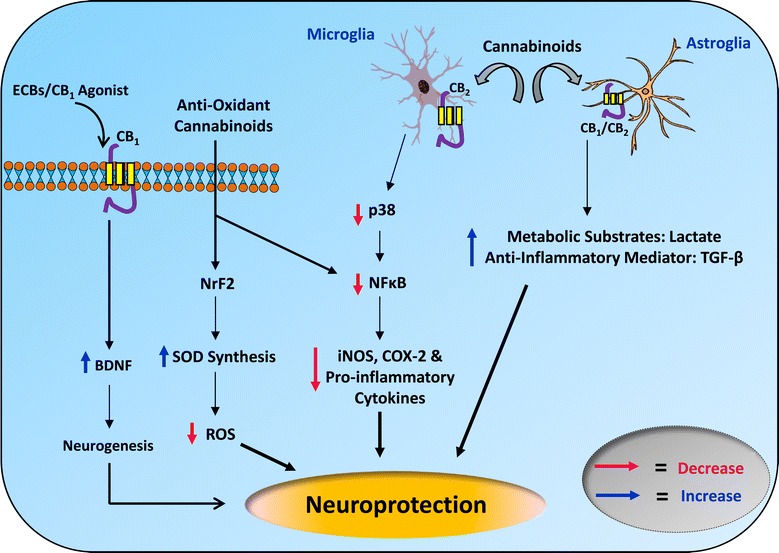
**Probable mechanisms to describe the neuroprotective (independent of the CB1 receptor) action of cannabinoids in PD.** Abbreviations: CB1, cannabinoid receptor 1; CB2, cannabinoid receptor 2; BDNF, Brain derived neurotrophic factor; ECBs, Endocannabinoids; ROS, reactive oxygen species; SOD, superoxide dismutase; NrF2, nuclear factor erythroid 2-related factor 2; NFκB, nuclear factor kappa-B; p38, p38 mitogen-activated protein kinases; iNOS, inducible nitric oxide synthase; COX-2, cyclooxygenase-2; TGF-β, transforming growth factor beta.

Supplementary mechanisms related to the direct improvement of endogenous antioxidant enzymes involve the activation of the anti-oxidant transcription factor nuclear factor erythroid 2-related factor 2 (Nrf-2) [8]. In a recent report, cannabidiol was found to up-regulate the transcription of Nrf-2 in BV-2 microglial cells [271]. Cannabidiol also increased the subsequent downstream enzymes including heme oxygenase-1, glutathione S-transferase, glutathione S-transferase peroxidase, NAD(P)H:quinone oxidoreductase and glutamate-cysteine ligase, which play central role in providing defence against cytotoxic and electrophile-induced oxidative stress [272]. However, more robust data are needed to support the antioxidant capacity of cannabinoids (Figure 2) in animal models. Nonetheless existing evidences does suggest that cannabinoids may serve as promising antioxidant therapy for treating PD.

### Therapeutic role of cannabinoids in neuroinflammation in PD

Neuroinflammation is a crucial pathological factor responsible for the demise of dopaminergic neurons in PD. Glial cells play a key role in neuroinflammation; elevated levels of activated microglia are found in the substantia nigra of patients with PD compared to brains of control subjects [90]. Lipopolysaccharide administration to mice increases CB2 receptor expression in nigral cells, and stimulation of these receptors shelters dopaminergic neurons from microglia-induced inflammation [273]. A limited amount of research has been performed pertaining to the anti-inflammatory properties of cannabinoids, but recent evidence substantiates that some cannabinoids, predominantly CB2 receptor ligands, may attenuate the neuroinflammation associated with PD [82,103,269,274]. Lesioned sites are observed to express CB2 receptors on astroglial cells [234]. Consequently, it is likely that these receptors augment the generation of neurotropic factors or metabolic substrates, such as lactate and ketone bodies, but these prospects has not been established yet. In a recent study utilizing intracerebral injection of 6-OHDA to induce parkinsonian symptoms in rats showed that the agonistic activity at CB2 receptors may offer neuroprotection. In this study, the 6-OHDA neurotoxin was injected into the medial forebrain bundle of rats, causing a substantial decline in the number of catecholaminergic neurons and inducing neuroinflammation [275]. In another study, daily pre-treatment of rats with 9-THC and cannabidiol for 2 weeks, followed by 6-OHDA injection, abated loss of dopaminergic neurons [225]. Using comparable *in vivo* methods, Garcia et al. established that treatment with synthetic the CB2 receptor agonist HU-303 displayed neuroprotective effects [269]. Transgenic rodents overexpressing the CB2 receptor were observed to mitigate demise of dopaminergic neurons and alleviate motor impairment following experimentally induced PD [113]. Similarly, injection of WIN-55,212-2 to rats for 4 weeks was found to decrease the number of activated microglia in the hippocampus and dentate gyrus as well as to reduce the mRNA levels of the proinflammatory cytokine IL-6 [276].

Cumulatively, these results indicate that activating CB2 receptors may decrease inflammation and avert neuronal death in the 6-OHDA model of PD. Investigations pertaining to the effects of cannabinoids on survival of isolated neuronal cells after 6-OHDA treatment revealed that the effects are mediated and governed by microglia [225]. Numerous CB receptor agonists, including 8-THC and 9-THC, decrease discharge of the proinflammatory cytokines tumor necrosis factor-α (TNF-α) and interleukins by human monocytes [103]. Additionally, WIN-55,212-2, cannabidiol and JWH-133 (CB2 receptor selective agonist), were all reported to decrease the ATP-induced rise in intracellular Ca^2+^ concentration in the N13 microglial cell line [277]. The effects of JWH-133 and WIN-55,212-2 were totally reversed by the selective CB2 antagonist, SR 144528 indicating a CB2 receptor dependent effect. However this antagonism was not observed in cannabidiol treated cells, indicating that CB2-independent mechanisms may also be favorable [277]. This CB2-independent mechanism involves the official pathway for the anti-inflammatory effects of most cannabinoid agonists [239]. Anti-inflammatory effects of cannabidiol are also associated with the regulation of microglial cell migration [104] and toxicity that stimulates production of pro-inflammatory mediators by these cells [274]. Cannabidiol interferes with transcription of NF-κB signaling and thereby controls the effectors genes contributing to inflammatory enzymes such as inducible NOS [274,278]. This NFκB inhibitory signaling mechanism may occur by decreasing p38 MAPK phosphorylation and hence avoiding its nuclear translocation to prompt expression of proinflammatory genes [278]. It has also been hypothesized that cannabidiol might bind peroxisome proliferator-activated receptor gamma [279,280], which antagonizes the action of NF-κB and thereby decreases the expression of pro-inflammatory enzymes, proinflammatory cytokines, and metalloproteases [281,280]. Although this proposed mechanism demands further study and confirmation, cannabidiol may elicit its anti-inflammatory effects by stimulating nuclear receptors and modulating their downstream targets (Figure 2). All these studies suggest that use of CB2 agonists [82,103] may mitigate neuroinflammation by modulating the activities of microglial cell which have been implicated in the pathogenesis of PD [41,82,282].

### Therapeutic role of cannabinoids in excitotoxicity in PD

The theory of excitotoxicity has long been applied to PD. Studies have confirmed that parkin controls the stability and function of excitatory glutamatergic synapses. Postsynaptic expression of parkin inhibits excitatory synaptic transmission and results in a marked loss of excitatory synapses in hippocampal neurons. In contrast, a deficiency of endogenous parkin or expression of parkin mutants linked to PD strongly improves synaptic efficiency and activates glutamatergic synapses. This activation is related with increased susceptibility to synaptic excitotoxicity [283]. The resulting excess glutamatergic transmission could be a source of excitotoxicity in the substantia nigra. In addition, continuous stimulation of NMDA receptors increases intracellular calcium levels and produces uncontrolled shifts in sodium, potassium, and calcium concentrations that disrupt ionic homeostasis and lead to severe cell swelling and cell death in PD [284,285]. Recently scientific researchers also explored the role of WIN-55,212-2 in dopaminergic neuronal death induced by a proteasomal synthase inhibitor (PSI) as well as its modulatory function in cytoplasmic accumulation of parkin and α-synuclein. WIN-55,212-2 was observed to protect PC12 cells from PSI-induced cytotoxicity by impeding PSI-induced poly-ADP ribose polymerase expression and activation of caspase-3. On the other hand WIN-55,212-2 was also found to decrease the cytoplasmic accumulation of parkin and α-synuclein. However in contrast to earlier report [286], where CB1 receptor agonist was documented to inhibit NF-κB activity; WIN-55,212-2 in combination with PSI was observed to potentiate the nuclear translocation of NF-κB in PC12 cells. This data indicates that WIN-55,212-2 modulates apoptosis induced by proteasomal inhibition, partly by regulating NF-κB activity. Lately it was documented that WIN-55,212-2 requires the presence of histidine triad nucleotide-binding protein 1 (HINT1) protein to neutralize the toxic effects of NMDA receptor mediated NO production and zinc release. This report hints towards HINT1 as a novel protein required for cannabinoid mediated protection against NMDA receptor-induced brain damage [287]. Apart from typical cannabinoid agonist and antagonists, FAAH inhibitors are also important to stimulate CB1 signaling mediated neuroprotection since, they are devoid of psychotropic effects associated with CB1 agonist [288]. In line with this reports, AM5206, a novel class of reversible FAAH inhibitor was investigated and demonstrated to provide neuroprotection *in vitro and in vivo*. In this study, AM5206 was found to protect pre and postsynaptic proteins in kainic acid induced excitotoxic damage to cultured hippocampal slices and rats [289]. This report supports the idea wherein ECBs provide protection against acute excitotoxicity [290]. In a related study, treatment with WIN-55,212-2 was found to inhibit TNF-α-induced rise in α-amino-3-hydroxy-5-methyl-4-isoxazolepropionic acid (AMPA) receptors and excitotoxicity in hippocampal cultures [291].

The prominence of CB1 receptors has been established in circumstances of excitotoxicity, such as those in rats lesioned with the excitotoxin quinolinate in the striatum [292]. Reducing glutamate release is a prominent effect of cannabinoid agonists that might affirm their role as potential anti-excitotoxic compounds for use as PD therapeutics (Figure 1) [57,293,294]. This effect has been verified both *in vitro*, using neuronal cultures from spinal cord [295] or from various brain regions [296], and *in vivo* using rodent models to induce ischemia [297]. Activating presynaptically located CB1 receptors with a cannabinoid agonist on glutamatergic terminals of subthalamonigral neurons leads to decreased glutamate release. This inhibitory effect of cannabinoid agonists on glutamate release is reversed by the selective CB1 receptor antagonist SR-141716 [298]. As CB1 receptors are expressed on the synapses of two opposing (glutamatergic/excitatory and GABAergic/inhibitory) neuronal populations, hence the activation of one and/or another receptor population may possibly induce dissimilar effects [299]. Despite various reports, precise mechanism for CB1 mediated neuroprotection is unknown. Lately, it was demonstrated that only restricted population of CB1 receptors located on glutamatergic terminal are necessary to protect neurons against excitotoxicity [299]. In context with these findings recently it was found that cannabinoid receptor interacting protein (CRIP1a) alters the neuroprotective ability of WIN-55,212-2 in cortical neurons exposed to glutamate by acting as a cannabinoid antagonist rather than an agonist [300]. Some selective cannabinoids, such as anandamide and dexanabinol (HU-211), directly act on NMDA receptors [293]. The neuroprotective activity of dexanabinol originates from its ability to directly inhibit the glutamate system by antagonizing the NMDA receptor at a site close to, but different from that of glutamate, glycine, MK-801 and phencyclidine [293,301]. Depending on this antagonistic ability, HU-211 directly diminishes NMDA-mediated Ca^2+^ influx into neurons [302]. Although dexanabinol also offers neuroprotection [303] and reduces the levels of TNF-α [234], because of it is antioxidant properties. Anandamide directly acts with NMDA receptors in cortical, hippocampal and cerebellar slices and decreases the NMDA-mediated calcium response [304]. This effect is independent of its neuroprotective effects mediated by activation of cannabinoid receptors. As cannabinoids have a well-known ability to inhibit glutamate release from subthalamonigral neurons [305], CB1 receptor agonists may also be useful to improve tremors observed in PD [306]. However, a clinical study carried out to confirm the effects of cannabinoids on parkinsonian tremor did not reproduce this preclinical finding [307].

### Therapeutic role of cannabinoids in neurogenesis in PD

Neurogenesis is the physiological process in which new neurons are generated and incorporated into the brain. Loss of neurite is one of the cardinal features of neuronal injury. Regulation of neurogenesis is stringently orchestrated by a number of different elements such as trophic factors, neurotransmitter systems and inflammatory cytokines. The establishment of new neurons and their connections is very essential for supporting normal neuronal function in neurodegenerative disorders where neurogenesis is impaired [308,309]. Neuroimmune networks and the brain ECB system have been closely associated to the process of adult neurogenesis [308,310]. For instance, genetic deletion of CB1 receptors leads to defective neurogenesis [311]. Also, they have been implicated in proliferation and neurogenesis of neural precursors in a model of excitotoxicity [312]. CB1 and CB2 receptors along with diacylglycerol lipase α have been identified in neural stem cells (NSC) where they govern and repair the proliferation of precursor cell in neurogenesis [313,314]. CB2 receptor has also been recognized as a physiological supervisor of proliferation in neural progenitor cells (NPC) [114]. In addition to CB1 and CB2 receptors, neurogenesis and expression of BDNF is also regulated at cellular level by ECB system. Endocannabinoids are observed to subdue neuroinflammatory processes contributing to the development of brain ageing as well as to the pathogenesis of neurodegenerative diseases [315]. Recently in animal models and postmortem studies of PD, adult neurogenesis was significantly affected, although the precise mechanisms and effects of these deviations are not yet fully understood [316]. Chronic neuroinflammation [317], mutant α-synuclein and aging have been demonstrated to decrease neurogenesis in the various stages of PD [318-320]. These findings are predominantly exciting, as they increase the probability of a role for endocannabinoids in protecting degenerated neurons in PD. Few studies have shown that, stimulation of CB1 receptor increases levels of BDNF [321] a neurotrophin required for adult neurogenesis [322] that is decreased in the PD brain [323,324]. In a mutual way, BDNF was also observed to increase neuronal sensitivity to 2-AG and noladin ether in cultured cerebellar granule neuronal cells [325]. Additionally, stimulation of CB1 receptor-mediated neurogenesis also acts through antagonizing the antineurogenic effect of NO [276,326].

Lately, it has been suggested that the diacylglycerol lipase-CB2 pathway by controlling neurogenesis in subventricular zone might help to reverse the decline in neurogenesis that occurs during aging [327]. In line with this report, age-linked decrease in neurogenesis was partially reinstated by treatment with WIN-55,212-2 in male F-344 rats [276]. On another hand, pharmacological agonism at CB2 receptor and inhibition of FAAH can also stimulate neurogenesis in the adult mouse [328]. New studies have discovered that NPC express CB1 and CB2 receptors. While stimulation of downstream PI3K-AKT-mTOR complex 1 signalling pathway along with CB2 receptors by endocannabinoids and non-psychoactive cannabinoids controls the extension of the NPC pool *in vitro* and *in vivo* [114,329]. Stimulation of cannabinoid receptors also is documented to suppresses chronic inflammatory responses through the reduction of pro-inflammatory mediators [314]. In a very recent experiment, pharmacological antagonism of CB1 and/or CB2 receptors eliminated or reduced proliferation of NSC. Thus indicating a vital role for both CB1 and CB2 receptors in the proliferation of NSC via IL-1 signaling pathway, which may be of therapeutic interest in the developing field of brain repair [314]. Specific agonism of CB1 receptors by ACEA displayed neuronal differentiation and maturation of NSC. Although treatment with CB2-specific agonist; JWH133 was futile to achieve a similar profile [330]. In contrast to this study, CB2-specific agonist, AM1241 was observed to stimulate differentiation of NPCs to neuronal cells. Also, administration of AM1241 to GFAP/Gp120 Tg mice lead to escalation of *in vivo* neurogenesis in the hippocampus as confirmed by growth in neuroblast and neuronal cells. Furthermore, treatment with AM1241 diminished astrogliosis and gliogenesis in GFAP/Gp120 Tg mice [331]. In conclusion, targeting ECB system at specific and selective level in the future might help to replenish and sustain the neural functions in PD.

## Conclusion and future directions

Numerous investigations have supported the observation that significant modulation of the cannabinoid signaling system occurs in PD. This conception has been reinforced by different electrophysiological, anatomical, and pharmacological findings. Therefore, pharmacological modulation of this this system with compounds that selectively target different elements of cannabinoid signaling may improve anomalies of motor behavior and provide neuroprotection. As cannabinoid-mediated functions do not control the cell activity directly coupled with their limited involvement in controlling brain functions, targeting the cannabinoid system may provide desired benefits in PD. Regardless of the experimental data, considerable number of issues need to be addressed, such as how, when, and where the ECBs function and how targeting ECB signaling could be a therapeutic advantage in PD. The important issues related to PD are motor disability and progressive nigral cell death. Despite existing therapies, no effective therapeutic intervention alleviates motor disabilities or provides neuroprotection. In contrast, substitute therapies for levodopa cause dyskinesia. Data in the past suggests that novel compounds with potent and specific activity for CB1, CB2, and TRPV1 receptors can be considered for treating PD. Compounds that selectively antagonize CB1 and perhaps TRPV1 receptors, may improve the motor disabilities such as bradykinesia and LID. The antioxidant mechanisms of certain cannabinoid compounds, which are independent of their cannabinoid receptor activity, also have a potential to be developed as a therapeutic PD. A second feature worthy of additional experimentation would be to elucidate the function and subsequent therapeutic potential of CB2 receptors in PD, as CB2 receptor agonists have curbed the inflammatory response incited by microglia in PD.

However it is recommended to develop antioxidant cannabinoids that lack CB1 receptor activity, as compounds with a CB1 interaction show deleterious effects on mitochondrial biogenesis and may exacerbate PD pathology [332]. Similarly, cannabinoids also have high abuse potential and cause dependence [333]. Therefore, drugs that modulate ECB levels by preventing their metabolism (FAAH and MAGL inhibitors) are therapeutically attractive targets, as they seem to have low or no abuse liability [333]. If these discoveries of cannabinoid-mediated suppression of inflammation and alleviation of motor symptoms are translated from preclinical to clinical systems, then cannabinoids might seem to be encouraging therapeutic for PD. However, emerging contradictory results from animal models simulating PD demand development of new pharmacological tools, improved screening models, upgraded technologies, and specific ligands for evaluating the therapeutic potential of cannabinoids in PD. Most studies investigating the therapeutic potential of cannabinoids in PD have been conducted in animal models, and an insufficient number of clinical trials have been carried out. Therefore, the therapeutic benefits demonstrated in animal models need more clinical evidence. Another challenge in PD is to develop a multifunctional or broad-spectrum cannabinoid that could decrease existing motor abnormalities, oxidative stress, and microglial activation seen in PD. To conclude, development of safe, effective cannabis-based medicines targeting different mechanisms may have a significant impact in PD therapy.

## Abbreviations

2-AG: 2-arachidonoyl glycerol
6-OHDA: 6-hydroxy dopamine
ALS: Amyotrophic lateral sclerosis
AD: Alzheimer’s disease
AMT: Anandamide membrane transporter
AIM: Abnormal involuntary movements
AMPA: α-amino-3-hydroxy-5-methyl-4-isoxazolepropionic acid
BDNF: Brain derived neurotrophic factor
COX-2: Cyclooxygenase 2
CaMKII: Calcium/calmodulin-dependent protein kinase II
DRG: Dorsal root ganglion
ECB: Endocannabinoid
FAAH: Fatty acid amide hydrolase
GABA: Gamma-aminobutyric acid
GPe: External segment of Globus pallidus
GPi: Internal segment of Globus pallidus
HD: Huntington’s disease
iNOS: Inducible nitric oxide synthase
LID: Levodopa-induced dyskinesia
LTD: Long-term depression
LTP: Long-term potentiation
MAGL: Monoacylglycerol lipase
MSNs: Medium spiny neurons
MS: Multiple sclerosis
MPTP: 1-methyl-4-phenyl-1,2,3,6-tetrahydropyridine
MAPK: Mitogen activated protein kinase
NO: Nitric oxide
NAPE-PLD: N-acyl phosphatidylethanolamine-specific phospholipase D
Noladin ether: 2-arachidonoyl glyceryl ether
Nrf-2: Nuclear factor erythroid 2-related factor 2
NPC: Neural progenitor cells
NSC: Neural stem cells
OEA: Oleoylethanolamide
O-arachidonoyl ethanolamine: Virodhamine
PD: Parkinson’s disease
PSI: Proteasomal synthase inhibitor
ROS: Reactive oxygen species
RGS4: Regulator of G-protein Signaling 4
SNpc: Substantia nigra pars compacta
SNpr: Substantia nigra pars reticulata
STN: Subthalamic nucleus
TNF-α: Tumor necrosis factor-α
